# Supplementary material for: The protein phosphatase-2A subunit PR130 is involved in the formation of cytotoxic protein aggregates in pancreatic ductal adenocarcinoma cells
Source: Cell Commun Signal. 2024 Apr 3;22:217. doi: 10.1186/s12964-024-01597-8 (PMC10993613; doi:10.1186/s12964-024-01597-8)

Supplemental Material for

The protein phosphatase-2A subunit PR130 is involved in the formation of cytotoxic protein aggregates in pancreatic ductal adenocarcinoma cells

Alexandra Nguyen[1], Al-Hassan M. Mustafa[1,2], Alessa K. Leydecker[1], Melisa Halilovic[1], Janine Murr[3], Falk Butter[4,5], and Oliver H. Krämer[1,*]

Correspondence to: okraemer@uni-mainz.de

This file includes:

Figures S1 to S5

Nguyen, Supplementary Fig. 1

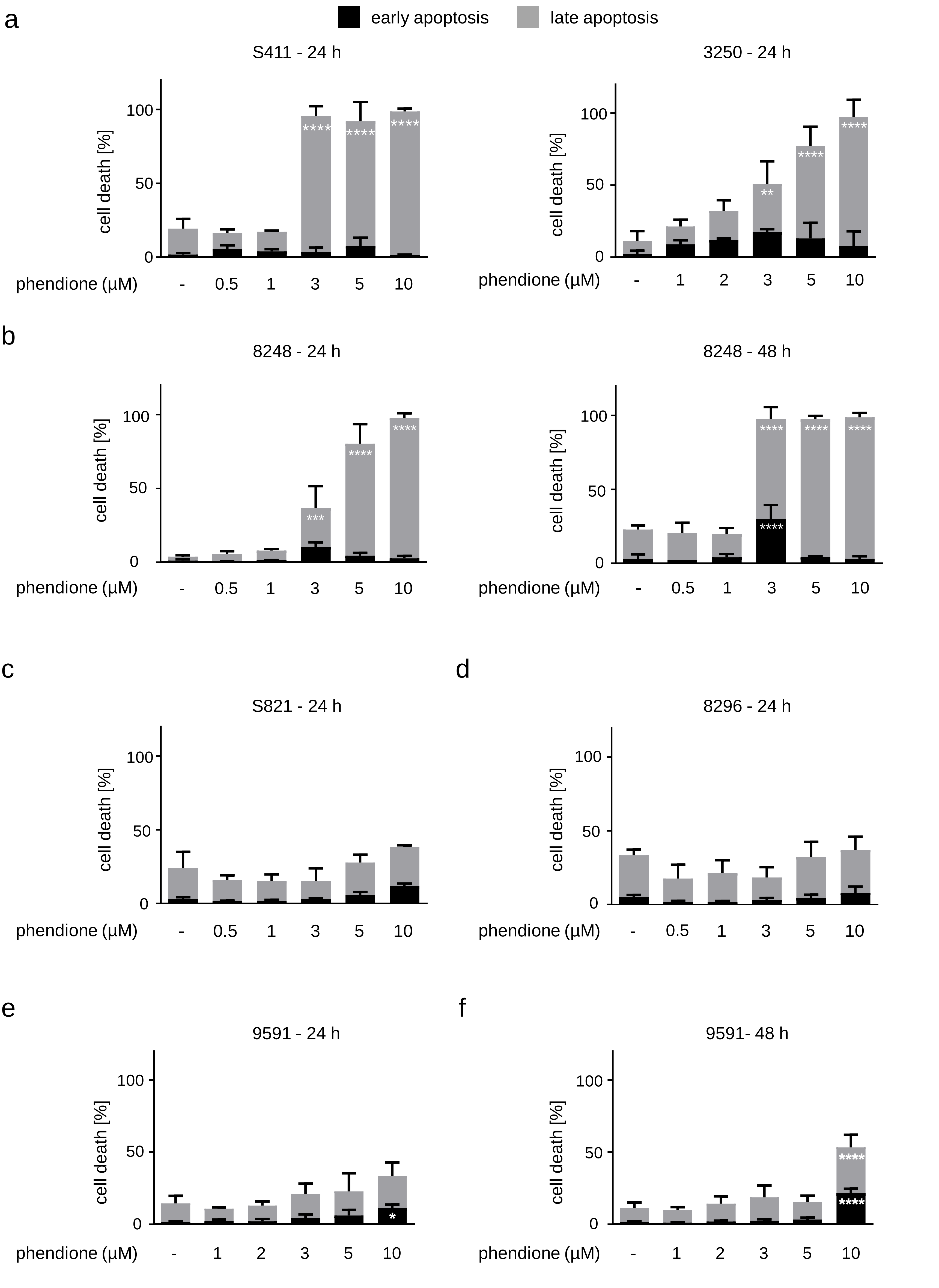

Nguyen, Supplementary Fig. 1

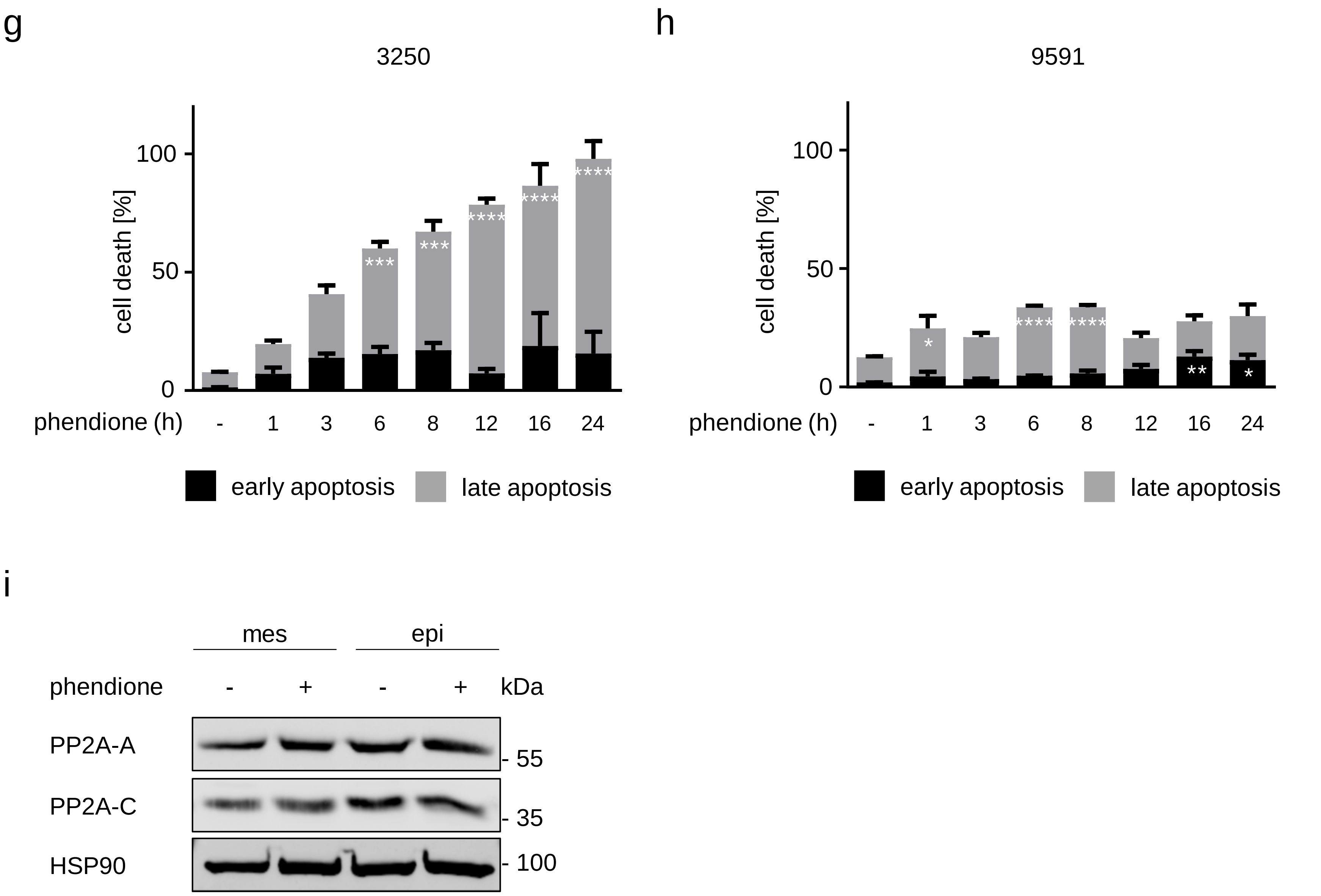

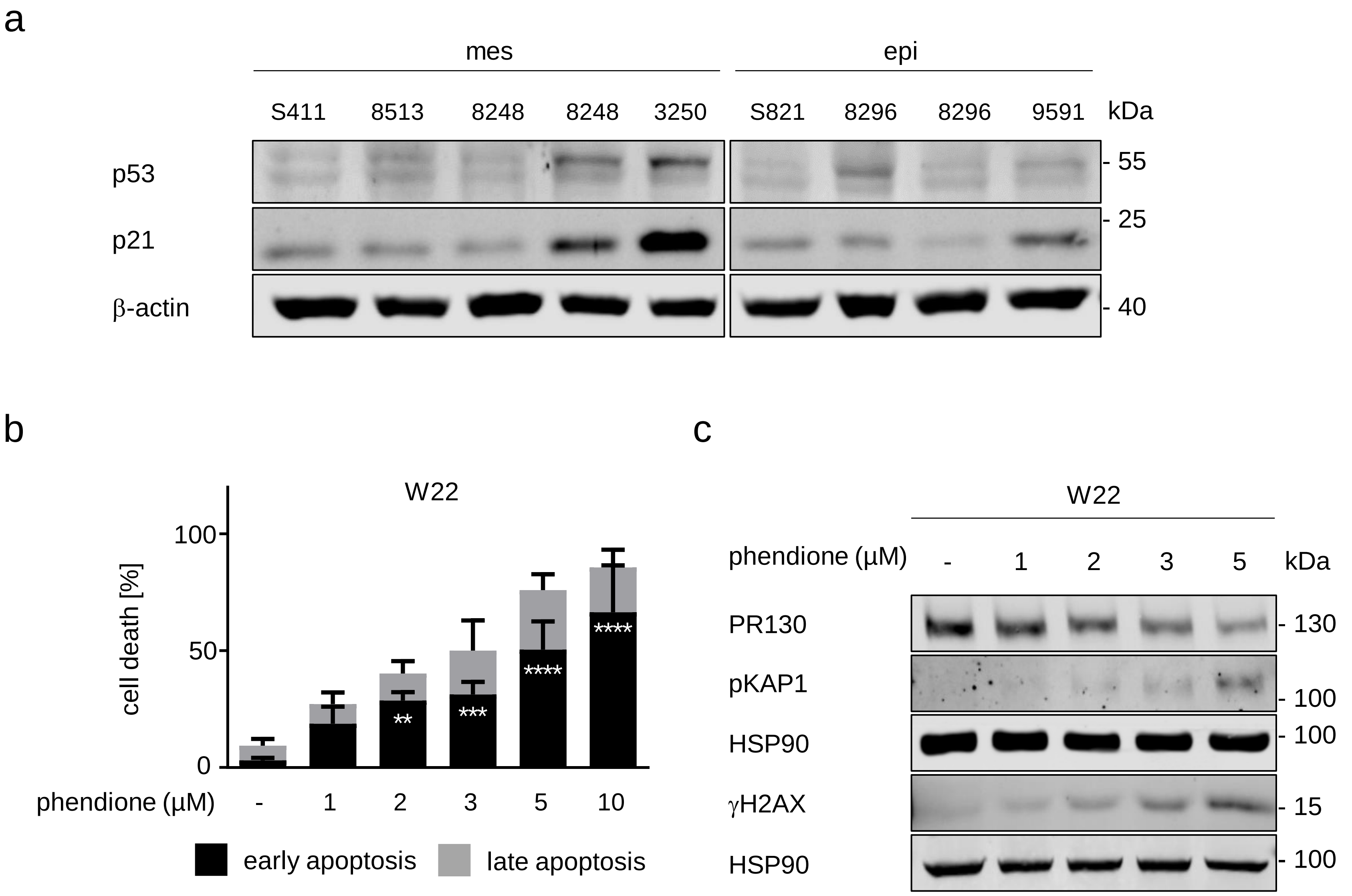
a
mes
epi
S411
8513
8248
8248
3250
S821
8296
8296
9591
kDa
p53
p21
β-actin
- 55
- 25
- 40
b
W22
cell death [%]
100
50
0
phendione (µM)
-
1
2
3
5
10
**
***
****
****
early apoptosis
late apoptosis
c
W22
phendione (µM)
-
1
2
3
5
kDa
PR130
pKAP1
HSP90
γH2AX
HSP90
- 130
- 100
- 100
- 15
- 100

Nguyen, Supplementary Fig. 3

a

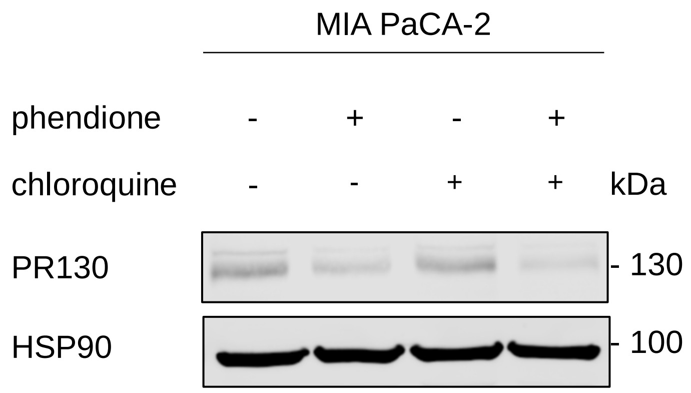

Nguyen, Supplementary Fig. 4

a

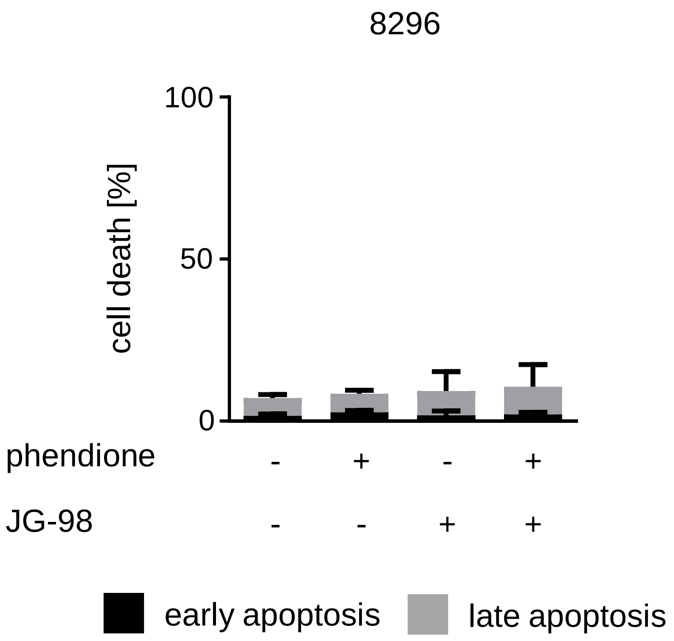

b

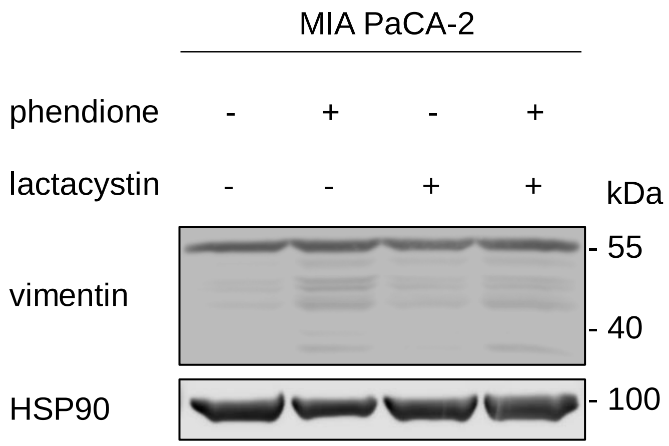

Nguyen, Supplementary Fig. 5

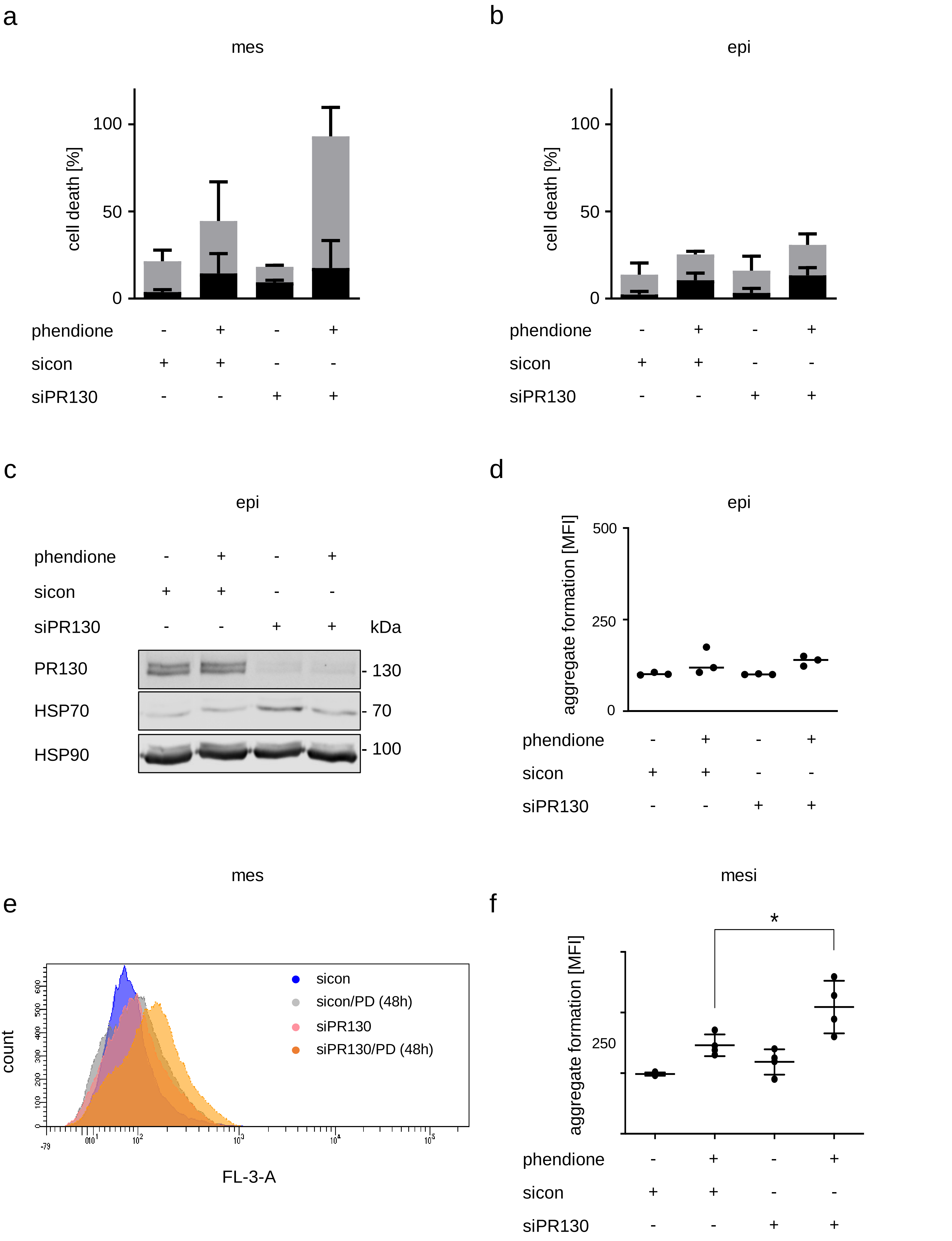

Supplement: Supplementary file 1 — Supplementary Material 1. [file 12964_2024_1597_MOESM1_ESM.zip › Nguyen_Supplemental figures_REVISION.pdf]
